# The implied motion aftereffect changes decisions, but not confidence

**DOI:** 10.3758/s13414-021-02331-z

**Published:** 2021-08-24

**Authors:** Regan M. Gallagher, Thomas Suddendorf, Derek H. Arnold

**Affiliations:** School of Psychology, The University of Queensland, Brisbane, QLD Australia

**Keywords:** Perceptual aftereffect, Decision-making, Sensory adaptation, Confidence

## Abstract

Viewing static images depicting movement can result in a motion aftereffect: people tend to categorise direction signals as moving in the opposite direction relative to the *implied* motion in still photographs. This finding could indicate that inferred motion direction can penetrate sensory processing and change perception. Equally possible, however, is that inferred motion changes decision processes, but not perception. Here we test these two possibilities. Since both categorical decisions and subjective confidence are informed by sensory information, confidence can be informative about whether an aftereffect probably results from changes to perceptual or decision processes. We therefore used subjective confidence as an additional measure of the implied motion aftereffect. In Experiment 1 (implied motion), we find support for decision-level changes only, with no change in subjective confidence. In Experiment 2 (real motion), we find equal changes to decisions and confidence. Our results suggest the implied motion aftereffect produces a bias in decision-making, but leaves perceptual processing unchanged.

## Introduction

An outstanding question in perception research is whether our thoughts, desires, emotions, or cognitions can change how our sensory systems operate. Usually, perceptual aftereffects are quantified by giving people prolonged and repeated exposure to a specific stimulus, and then measuring changes in response to a range of stimulus intensities (e.g. the brightness of lights, or the volume, pitch or frequency of a tone; see Clifford et al., 2007, for a review). Aftereffects are also typically constrained to a common sensory dimension, such as when a moving adaptor influences the perceived motion of a test (Barlow & Hill, 1963). However, recent research has started revealing aftereffects in which test stimuli are only conceptually related to the adapting stimulus. Such a method offers an empirical approach to determine if high-level cognitions (such as extracted meaning) can filter down to change perception.

**Fig. 1:**
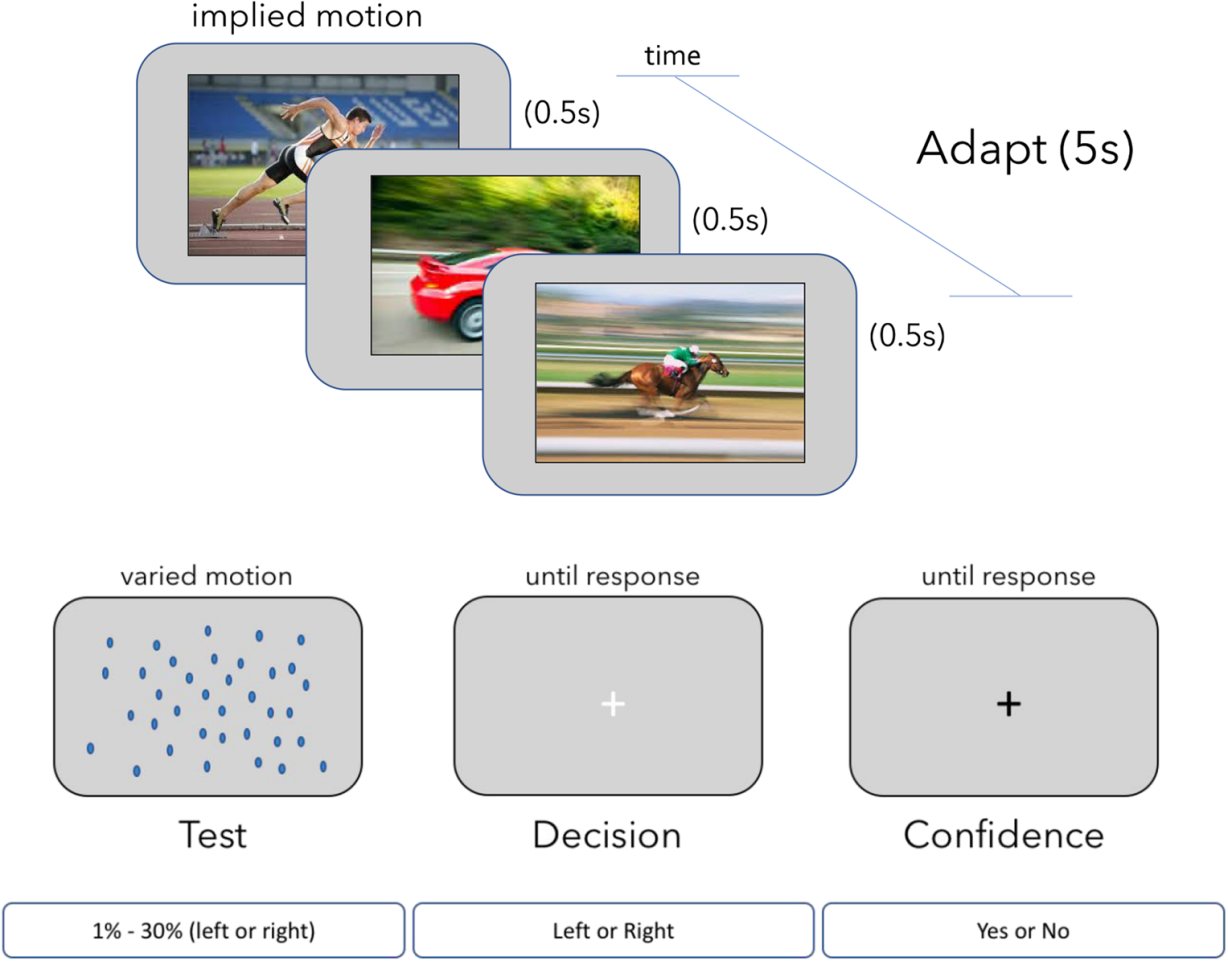
Experiment procedure. In Experiment 1, participants adapted to still photographs that depicted motion to either the left or right (one direction for each testing block). Adapting stimuli appeared for 18 s on the first trial of each block, and on the middle trial, and for 6 s on all other trials. There was no adaptation period in Experiment 2. Each trial then had a dot test probe. Tests were present for 1 s, appearing on the second frame after the adapting stimulus disappeared. A new trial began once participants had recorded their direction decision (left or right) and reported their confidence in their decision (yes or no)

One study conducted by Winawer et al. (2008) had participants adapt to still photographs that *implied* motion (either leftward or rightward, or inward or outward; see Fig. 1a). In a second study (Winawer et al., 2010), participants adapted to a static grating and were asked to *imagine* that it was moving. In both studies participants then judged the motion direction of a dynamic dot stimulus. In each case the adaptation phase (static images, or imagined motion) gave rise to a negative aftereffect: participants had an increased probability of judging a (possibly ambiguous) stimulus as having moved in the opposite direction. This pattern of results is broadly consistent with the classic motion aftereffect (Barlow & Hill, 1963), and the authors concluded that the adaptation task had directly changed motion perception. While this interpretation is intuitive, there is an equally plausible interpretation: viewing static images that imply movement, or imagining movement, might engage motion-related cognitions that bias categorical decisions, but leave the sensory processes underlying motion perception unchanged (for a related discussion of these issues, see Yarrow et al., 2011).

Recent research suggests that subjective confidence reports can provide an important additional source of information to help discern whether it is probable that an aftereffect has a perceptual basis (Gallagher et al., 2019). When decisions are changed by sensory adaptation, both the decision and confidence functions can provide equivalent measures of the perceptual aftereffect. However, when decisions change for reasons other than a perceptual change (e.g. due to a biased pattern of responses when inputs are ambiguous), the range of inputs that elicit low confidence in categorical judgments can remain unchanged, but a dissociation between decision and confidence response profiles can emerge because participants commit to systematically biased categorisations whenever inputs are ambiguous. In this scenario, the dissociation of categorical decisions from subjective confidence constitutes evidence for the aftereffect being driven, at least in part, by non-perceptual decision processes.

## Perceiving versus deciding

Changes in decision-making usually constitute acceptable evidence for sensory adaptation. However, decisions can change in the absence of perceptual changes (see, e.g., Morgan et al., 2012). Based on biased decisions alone, we cannot tell if a given aftereffect has resulted from changes to perception or from changes to a decision process independent of perception (for greater discussion on this topic, see Firestone & Scholl, 2016; Fritsche et al., 2017; Morgan et al., 2012; Storrs, 2015).

Sensory evidence consists of the physiological information made available to the brain by the sense organs. Sensory evidence is typically considered to be contaminated by stochastic Gaussian noise, so different sensory information can be encoded on a trial-by-trial basis, even if people are repeatedly exposed to an unchanging physical input. Decision criteria can be regarded as a boundary, or a threshold value, separating when people will decide whether to classify a stimulus as belonging to one category or another (e.g., as moving to the left or right). The interpretation of weaker stimuli, with encoded values close to a decision boundary, can be shifted to either side of the boundary on a trial-by-trial basis by sensory noise, by a changing decision criterion, or both. As a result, decisions made about weak signals are often probabilistic. Stronger signals might be equally variable from trial-to-trial, but variable decision or sensory processes will have less influence on their interpretation, because the encoded value is more distant from the criterion.

As an example, if an observer is required to choose between two alternatives when an input is perceptually ambiguous, they may rely on higher-order cognitive (i.e. non-perceptual) aspects of decision-making. One might simply guess, leading to an equal likelihood of choosing either option. Alternatively, they might adopt some other strategy that carries a greater bias toward one category or another. A decision strategy could manifest as either a negative or a positive bias (or none), independent of sensory evidence and other post-perceptual processes (see Yarrow et al., 2011). Moreover, a wilfully adopted decision bias can change estimates of decision boundaries, without changing the precision of perceptual judgments (Morgan et al., 2012) or the central tendency of the associated distribution of confidence (Gallagher et al., 2019).

The ambiguity of perceptual categorisation data creates an opportunity for subjective confidence to be helpful in diagnosing the reason for a change in responding. Categorical decisions and confidence reports provide two sources of information that are each informed by sensory evidence. Confidence can, however, be dissociated from categorical decision making when a cognitive bias causes a change in responding (see Gallagher et al., 2019). We reason that this can happen when people make systematically biased category judgments about stimuli that elicit uncertainty. The range of inputs that elicit uncertainty are, however, unchanged – so confidence responding is unchanged. Measuring changes in confidence, in addition to category responses, could thus help to distinguish changes in perception from changes in decision-making. In the present study, we find evidence that viewing still photographs depicting movement changes categorisations of ambiguous inputs, but does not change confidence. On this basis, we argue that it is improbable that the implied motion aftereffect has a perceptual origin.

## Experiment 1

### Method

#### Participants

All participants (30 for each Experiment) were recruited from the University of Queensland’s Psychology department. Sample sizes of 30 were set for all Experiments, as these are comparable to samples used in the original studies of the imagined and implied motion aftereffects. Participants were drawn from a first-year student pool, who received course credit for their participation. All were naïve to the purpose of the experiments.

#### Ethics

Ethical approval for all experiments was obtained from the University of Queensland’s Ethics Committee, and experiments were conducted in accordance with committee guidelines. Each participant provided written informed consent, and were aware that they could withdraw from the study at any point without penalty.

#### Materials and stimuli

Stimuli were presented on a Dell LCD monitor (1,024 × 768 pixels). All computers were running Matlab software and the Psychophysics Toolbox (Brainard & Vision, 1997; Pelli, 1997). All monitors had a screen refresh rate of 60 Hz. Adapting stimuli (Experiment 1 only) and test stimuli were presented within an aperture size of 300 × 175 pixels against a grey background (RGB = 125,125,125).

Adapting stimuli were photographs taken from a Google Image search, after searching for images that depicted fast movement along the horizontal plane. The implied motion stimuli were then compiled into a set of 100 photographs, with an approximately equal aspect ratio in portrait orientation. The dimensions of photographs were re-sized before presentation, so that they were all equal in aspect ratio. Each photo was mirror-flipped, so it could be used to depict both directions. Test stimuli consisted of 100 dots rendered blue against a grey background. Each dot was 1 pixel in size. Initially, dots were drawn at random locations within the aperture window. Dot coherence values ranged from −30 (30% coherence leftward) through 0 (random motion) to +30 (30% coherence rightward). Test stimuli were set to one of 11 coherence values (−30 −20 −10 −6 −3 0 3 6 10 20 30), presented in a randomised order. Coherent motion was achieved by displacing coherent dots left or right by one pixel on successive frames. Coherently moving dots were selected at random on each frame, so no individual dot could be tracked across the screen. All other dots were redrawn at random locations.

#### Procedure

Participants sat comfortably in a chair approximately 55 cm from the display, resting their hands on the keyboard’s directional buttons while fixating a central cross-hair. If there was an adaptation phase, the adapting stimulus was presented for 18 s on the first trial for each of five blocks, and again on the middle trial when implied motion direction reversed. For all other trials the adapting stimulus lasted for five seconds. Each static image within an adapting sequence was presented for 0.5 s. Participants passively viewed implied motion images without responding. See Fig. 1 for a representation of the task procedure.

After the adaptation phase, participants were presented with one of the 11 dot-motion test probes. Tests were presented for one second before disappearing, leaving only the fixation cue. Participants reported whether the test had appeared to be moving left (by pressing the left arrow key) or right (by pressing the right arrow key). If participants could not determine the test direction, they were instructed to make their best guess.

Once the direction judgment had been made on each trial, the fixation cue turned black, prompting a confidence response. Participants indicated whether they felt high confidence in their direction judgment by pressing the up arrow on the keyboard, or low confidence (or that they were guessing) by pressing the down arrow. The fixation cross turned white once the confidence response had been provided, and a new trial started immediately. Each of the 11 stimulus values was tested five times per block, and participants completed two blocks in total (once with a leftward adaptor and once with a rightward adaptor) resulting in 110 individual test trials per participant. The initial adaptor direction was randomised for each participant.

#### Data preparation

Categorical direction decisions were scored as a 1 if the person said the stimulus moved to the right, or 0 if they said left. The response function approximated a cumulative Gaussian distribution when proportion of rightward decisions are plotted as a function of motion direction and coherence. The inflection point of functions (for unbiased observers) should approximate 0% coherence, indicating an equal probability of choosing leftward and rightward motion when the stimulus has random physical movements.

**Fig. 2:**
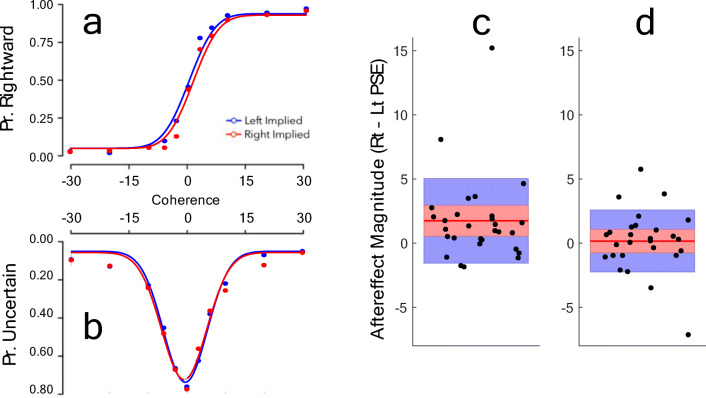
Results of Experiment 1. (**Left**) Psychometric functions fit to (**a**) the proportion of rightward responses and (**b**) the proportion of low-confidence responses, both as a function of motion direction and coherence, with data averaged across participants. Note that these data are illustrative, as reported data analyses relate to individual data sets. Fits to confidence data are shown inverted, as we felt it was more intuitive for low-confidence to be associated with a downward direction. Separate lines depict the direction of the implied motion adaptor. (**Right**) Average aftereffect magnitudes, as measured by decision (**c**) and confidence (**d**). Data points show individual aftereffect magnitude estimates. Red horizontal lines show average aftereffects, red shaded regions depict ±1 SEM, blue shaded regions ±1 SD

Confidence responses were scored as a 0 if the person reported a *high* confidence in their direction decision, and as a 1 if they expressed *low* confidence. Summed responses from each participant therefore provide a confidence distribution reflecting the proportion of trials on which the participant had experienced uncertainty (expressed as low-confidence). This approximates a raised Gaussian function when responses are plotted as a function of motion direction and coherence (see Fig. 2b). The peak of uncertainty functions is the point of *lowest* confidence, which should be centred around 0% coherence for an unbiased observer.

Two aftereffect measures were taken for each participant in each Experiment: one from differences between inflection points of cumulative Gaussian functions fit to decision responses, and one from differences in the peak of raised Gaussian functions fit to confidence responses. In all cases, when functions were fit to individual data we compared summed absolute residuals between function fits and data to summed absolute residuals between the mean response and proportional individual data points (see Fig. 2a, b). When participants respond randomly, individual data points cluster about the mean response, regardless of input. Accordingly, when summed absolute residuals from function fits were not *smaller* than summed absolute residuals between the mean response and the functions’ proportional individual data points, we excluded that participant’s data from further analysis. In Experiment 1, this resulted in the exclusion of data from three participants, and in Experiment 2 this resulted in the exclusion of data from seven participants.

### Results

All t-tests reported are two-way repeated-measures tests for equality of means. All Bayes Factor analyses were estimated using the bayes Factor toolbox for Matlab.

#### Implied motion aftereffect

Analyses for Experiment 1 showed that adaptation to still images depicting motion had a robust influence on direction decisions. Results showed that decisions following leftward adapting images (L_PSE_ = 0.53; SD = 2.62) were significantly different from decisions following rightward adapting images (R_PSE_ = 2.26; SD = 4.52; t(28) = 2.84, p = .010, 95% CI 0.48–2.99, Cohen’s *d* = 0.47; BF10 = 5.26). However, implied motion adaptation did not influence measures of confidence. The central tendency of confidence reports following leftward implied motion adaptors (L_CONF_ = 1.60; SD = 5.59) was not significantly different to the central tendency of confidence reports following rightward implied motion adaptors (R_CONF_ = 0.79; SD = 2.33; t(26) = 0.35, p = .730, Cohen’s *d* = 0.19; BF10 = 0.21). These data are depicted in Fig. 2.

## Experiment 2

### Method

The methodological details for Experiment 2 are identical to Experiment 1 except for the following. One, participants sequentially responded to the test stimuli without an adapting stimulus. Two, there were eight test values (−30 −15 −5 −1 +1 +5 +15 +30) rather than the 11 test values in Experiment 1. There was no 0% coherence value, which allowed us to analyse responses according to the physical direction of the previous test. Three, participants completed 50 observations of each of the eight test values for a total of 400 observations per observer.

### Results

#### Rapid motion aftereffect

**Fig. 3:**
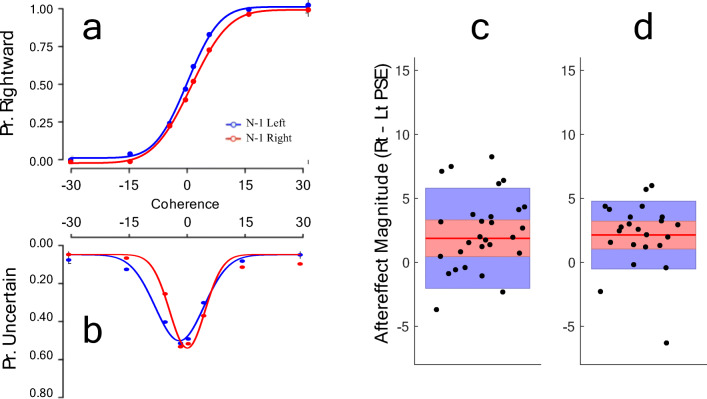
Results of Experiment 2. (**Left**) Psychometric functions fit to (**a**) the proportion of rightward responses and (**b**) the proportion of low-confidence responses, both as a function of motion coherence, with data averaged across participants. Note that these data are illustrative, as reported data analyses relate to individual data sets. Fits to confidence data are shown inverted. Separate lines depict fits to data split according to the *physical* direction of the previous test stimulus. (**Right**) Average aftereffect magnitudes, as measured by decisions (**c**) and confidence (**d**). Data points show individual aftereffect magnitude estimates. Red horizontal lines show average aftereffects, red shaded regions depict ±1 SEM, blue shaded regions ±1 SD

Analyses for Experiment 2 showed that the test motion direction on the previous trial had a robust influence on subsequent direction decisions. Results showed that decisions following leftward tests (L_PSE_ = -1.26; SD = 3.04) were significantly different from decisions following rightward tests (R_PSE_ = 0.80; SD = 3.17; t28 = 2.59, p = .015, Cohen’s *d* = 0.66, 95% CI 0.39–3.37, BF10 = 3.19). The previous test motion direction also changed measures of confidence. The central tendency of confidence reports following leftward tests (L_CONF_ = -1.17; SD = 1.6) was significantly different to the central tendency of confidence reports following rightward tests (R_CONF_ = 2.29; SD = 6.04; t(22) = 3.87, p = .001, 95% CI 0.99–3.28, Cohen’s *d* = 0.78; BF10 = 41.32). The measured effect of previous trials was not significantly different for decision (∆PSE = 1.88; SD = 3.91) and confidence reports (∆CONF = 2.13; SD = 2.65; t(22) = 0.30, p = .768, Cohen’s *d* = 0.08; BF10 = 0.20). These data are depicted in Fig. 3.

## Discussion

Our results suggest that the implied motion aftereffect is the result of changes to post-perceptual decision processes. Experiment 1 replicated the effect of viewing a stream of still photographs implying directional motion. As predicted, and consistent with previous research (Winawer et al., 2008), our results showed that participants more often reported that tests were moving in the *opposite* direction relative to the direction implied by static adapting images. The results of confidence responses, however, suggested the region of peak uncertainty was unchanged by adapting to implied motion.

Previous research has shown that perceptual changes can result in an equivalent influence on the central tendency of both decision and confidence reports (Anobile et al., 2020; Gallagher et al., 2019; Maldonado Moscoso et al., 2020), whereas changes in post-perceptual processes can change decision-making without changing confidence. Experiment 2 showed that the point of subjective equality (PSE) and the central tendency of confidence reports were equally influenced by the motion direction of the previous test stimulus. Tests on the current trial (trial n) were more often reported to be moving in the *opposite* direction relative to the last test (trial n-1). The probability that the current test would evoke low confidence was also influenced by the previous test stimulus. Importantly, the shift in confidence reports here was in the same direction as the shift in decisions, and of indistinguishable magnitude. These results are consistent with both responses (direction decisions and confidence) being informed by a common source of information (perception), which was equally influenced by recent sensory history.

### Does cognition penetrate perception?

Aftereffects induced by implying or imagining motion have previously been interpreted as resulting from a top-down influence of cognition on motion perception (Pavan et al., 2011; Winawer et al., 2008, 2010). This claim has been bolstered by electrophysiological evidence that viewing photographs depicting implied motion activates some of the same direction-selective cortical circuits as viewing real motion (Lorteije et al., 2006; Lorteije et al., 2007). However, it is not clear from either set of evidence that motion perception has been changed by cognition. Instead, different decisions could be reached, and similar neural activations could be triggered, by changes to decision processes, even in the absence of changes to motion perception.

The pattern of results in Experiment 2 is consistent with evidence for a *perceptual* aftereffect, whereas the decision aftereffect in Experiment 1 is consistent with changes in non-perceptual processes. Implied motion (Experiment 1) and serial dependence (Experiment 2) both changed motion direction decisions. However, only exposure to real motion produced a change in confidence. Our results therefore show no evidence for a cognitive penetration of perception triggered by still photographs that imply directional movement.

### Why do non-perceptual aftereffects have systematic directions?

Although our data cannot determine why implied motion adaptation produces a negative response bias, previous research demonstrates that arbitrary decision strategies can produce data consistent with either a positive or a negative aftereffect (Gallagher et al., 2019; Morgan et al., 2012). In our task, one possibility is that people surreptitiously perform a categorisation task whenever an unambiguous direction cue is encountered. This could result in a bias toward categorising ambiguous inputs in the *opposite* category, relative to the recently viewed (adapting) direction cue. This describes the frequency principle – a propensity to assign equal numbers of inputs to either category when making dichotomous classifications (Parducci et al., 1960; Parducci & Wedell, 1986). So, if a given class of input is repeatedly ‘implied’ by an adapting signal, people might compensate by assigning more weight to the less frequently encountered category. Our confidence data agree with an emerging picture in decision-making research, which describes adaptation of decisions as being potentially independent of perception (e.g., Witthoft et al., 2018), a finding that has previously included the implied motion aftereffect (see Mather & Sharman, 2015).

A participant whose task is to imagine or infer movement while viewing stationary objects might, when prompted, show a statistical preference for the unimagined direction when test stimuli are ambiguous (Winawer et al., 2010). In this example, the observer could be indexing unchanged sensory evidence against a different criterion when uncertain. Changes in decision-making could thus occur without physiological changes in motion-sensitive brain regions, due to a shift in decision criteria. This would alter the measurement of maximal categorical ambiguity – the inflection point of a cumulative gaussian function fit to binary categorical decisions. This measure is often referred to as the point of subjective equality (PSE), and changes to this metric from baseline are used to estimate aftereffect magnitudes. However, while a shift in a decision criterion will alter decision-making, they might have no influence on the range of inputs that elicit uncertainty. An additional important point is that both measures (decisions and confidence) are informed by sensory encoding. So, having people report on decisional confidence, in addition to committing to a categorisation, might reveal a dissociation. Confidence, or expressions of uncertainty, could remain veridical in the case where categorisations are biased solely by decisional processes.

### Our findings in relation to previous related research

A core feature of our serial dependence results is in agreement with some previous research, showing that perceived motion can undergo rapid sensory adaptation (Glasser et al., 2011; Kanai & Verstraten, 2005), and that rapid *negative* motion aftereffects have an equivalent influence on both decision and confidence reports (Gallagher et al., 2019). But while our serial dependence results suggest a negative aftereffect, some other studies of serial dependence have reported positive aftereffects (Cicchini et al., 2017; Fischer & Whitney, 2014; Fornaciai & Park, 2018).

Fischer and Whitney (2014), for instance, found that when people adjusted bar orientations to match the perceived orientations of test Gabors, settings were biased *toward* the physical orientation of previous tests. However, while Fritsche et al. (2017) replicated this finding when participants similarly matched bars to perceived test orientations, they found an *opposite* (negative) aftereffect when people were asked if pairs of test stimuli had been matched in orientation. Importantly, in this paradigm only one of each pair of test stimuli had been presented in the same location as a preceding oriented stimulus. These authors asserted that, of these tasks, the same/different categorisation that had delivered evidence for a negative aftereffect more probably reflected changes in perception than the delayed match-to-sample task – so they attributed the positive serial dependency to a non-perceptual cause (Fritsche et al., 2017). Cicchini et al. (2017) replicated these findings, with the caveat that they too found evidence for an attractive (positive) aftereffect when orientation differences between successive inputs were slight (<15°, see Fig. 1b in Cicchini et al., 2017). What are we to make of these findings?

We would suggest that the perceptual or non-perceptual status of serial dependencies between successive oriented inputs are uncertain, as to date causal attributions have relied on reasoned assertion rather than conclusive empirical evidence (Cicchini et al., 2017; Fischer & Whitney, 2014; Fritsche et al., 2017). Our results suggest these paradigms could benefit from the addition of confidence judgments in order to determine if the range of test stimuli that elicit uncertainty undergoes change – as we predict when an aftereffect has a perceptual basis.

### Limitations of our paradigm

While our general paradigm – reporting on confidence in addition to committing to perceptual categorisations – has benefits, it also has limitations. One is that our paradigm involves making two reports on each trial, as opposed to one. This presumably places a greater demand on visual short-term memory than asking participants to make a single report, and introduces a temporal confound based on the order of reporting. This could be mitigated by having people make combined responses (i.e. by committing to high-confidence left, to low-confidence left, low-confidence right or to high-confidence right responses), or by requiring just one type of response per trial, with different responses (decision or confidence) required on different trials, or in different blocks of trials. Experiments would be needed to see if different approaches impact performance levels to an extent that justifies adopting our chosen response mode.

Another obvious limitation is that confidence can be subject to a systematic bias, just as categorical decisions are. Indeed, evidence suggests confidence has at least two determining factors. One is a dynamic factor that reflects how well sensory information has been encoded from moment to moment (de Gardelle & Mamassian, 2014; Fleming et al., 2012; Keane et al., 2015; Spence et al., 2016). Another is a broader personality trait, less related to how well sensory information has been encoded (Kleitman & Lazar, 2007). This can be regarded as a bias factor for confidence judgments in our paradigm. In our second experiment, for instance, a strong bias to report high confidence regardless of input led to the exclusion of data from 4 participants.

The influence of an overall bias to only report high (or low) levels of confidence could be mitigated. Instead of forcing people to make high or low confidence ratings, they could instead be allowed to use a continuous scale anchored by oppositely signed (negative and positive) decisional categorisation values at the extremes, and passing through ‘guessing’ categorisations near the midpoint. To achieve similar analyses to those reported here, confidence data could be categorised by the experimenter relative to each individual’s median absolute level of confidence (with decisional data categorised according to the sign of the setting). Perfectly balanced non-categorical ‘guesses’ would not be allowed, to ensure a categorical decision could be measured from a setting along the continuous scale on each trial. Provided people display some systematic variance about their median level of confidence, this approach could obtain analysable confidence data from people who have an overall bias to only report high (or low) levels of confidence.

One positive feature of confidence biases, however, is that they do not tend to distort measures of central tendency, provided the participant makes at least some uncertain (and confident) responses. In the extreme, confidence data can be uninformative, if people always report having high or low confidence, but this tendency is not misleading in terms of central tendency – in contrast to any systematic bias when committing to decisions about perceptual categories.

## Conclusion

Our data suggest that the implied motion aftereffect reflects changes in decision-making, in the absence of changes to sensory processes. This contrasts with the motion aftereffect that changes both directional decisions and estimates of directional uncertainty.

## Data Availability

All data and analysis scripts associated with this publication will be made publically available via UQeSpace.

## Declaration

RMG and DHA designed the experiments. RMG conducted the experiments, analysed the data, and wrote the manuscript. RMG, TS, and DHA revised and approved the final manuscript.
